# SOD1 Is an Integral Yet Insufficient Oxidizer of Hydrogen Sulfide in Trisomy 21 B Lymphocytes and Can Be Augmented by a Pleiotropic Carbon Nanozyme

**DOI:** 10.3390/antiox13111361

**Published:** 2024-11-07

**Authors:** Karthik Mouli, Anton V. Liopo, Larry J. Suva, Kenneth R. Olson, Emily A. McHugh, James M. Tour, Paul J. Derry, Thomas A. Kent

**Affiliations:** 1 Center for Genomics and Precision Medicine, Institute of Bioscience and Technology, Texas A&M University Health Science Center, Houston, TX 77030, USA; kmouli@tamu.edu (K.M.); liopo@tamu.edu (A.V.L.); 2 Department of Chemistry, Rice University, Houston, TX 77005, USA; emily.mchugh@utexas.edu (E.A.M.); tour@rice.edu (J.M.T.); 3 Department of Veterinary Physiology and Pharmacology, School of Veterinary Medicine and Biomedical Sciences, Texas A&M University, College Station, TX 77843, USA; lsuva@cvm.tamu.edu; 4 Department of Physiology, Indiana University School of Medicine South Bend, South Bend, IN 46617, USA; kenneth.r.olson.1@nd.edu; 5 Department of Biological Sciences, University of Notre Dame, Notre Dame, IN 46556, USA; 6 Department of Materials Science and NanoEngineering, Rice University, Houston, TX 77005, USA; 7 Smalley-Curl Institute, Rice University, Houston, TX 77005, USA; 8 NanoCarbon Center and the Rice Advanced Materials Institute, Rice University, Houston, TX 77005, USA; 9 School of Engineering Medicine, Texas A&M University, Houston, TX 77030, USA; 10 Stanley H. Appel Department of Neurology, Houston Methodist Hospital and Research Institute, Houston, TX 77030, USA

**Keywords:** nanozyme, sulfide, Down syndrome, oxidative stress

## Abstract

Down syndrome (DS) is a multisystemic disorder that includes accelerated aging caused by trisomy 21. In particular, overexpression of cystathionine-β-synthase (CBS) is linked to excess intracellular hydrogen sulfide (H_2_S), a mitochondrial toxin at higher concentrations, which impairs cellular viability. Concurrent overexpression of superoxide dismutase 1 (SOD1) may increase oxidative stress by generating excess hydrogen peroxide (H_2_O_2_) while also mitigating the toxic H_2_S burden via a non-canonical sulfide-oxidizing mechanism. We investigated the phenotypic variability in basal H_2_S levels in relation to DS B lymphocyte cell health and SOD1 in H_2_S detoxification. The H_2_S levels were negatively correlated with the DS B lymphocyte growth rates but not with CBS protein. Pharmacological inhibition of SOD1 using LCS-1 significantly increased the H_2_S levels to a greater extent in DS cells while also decreasing the polysulfide products of H_2_S oxidation. However, DS cells exhibited elevated H_2_O_2_ and lipid peroxidation, representing potential toxic consequences of SOD1 overexpression. Treatment of DS cells with a pleiotropic carbon nanozyme (pleozymes) decreased the total oxidative stress and reduced the levels of the H_2_S-generating enzymes CBS and 3-mercaptopyruvate sulfurtransferase (MPST). Our results indicate that pleozymes may bridge the protective and deleterious effects of DS SOD1 overexpression on H_2_S metabolism and oxidative stress, respectively, with cytoprotective benefits.

## 1. Introduction

Down syndrome (DS) is the most common chromosomal abnormality in humans, affecting nearly 1 in 700 live births in the United States [1,2]. It is caused by the presence of an additional copy of chromosome 21, most commonly due to trisomy but also as a result of Robertsonian translocations and mosaicism caused by mitotic nondisjunction [1]. DS is associated with a spectrum of neurocognitive impairments, developmental delays, musculoskeletal anomalies, congenital heart defects and impaired immunity, with extensive variability in the presentation of clinical symptoms [3]. Prior studies by other groups have attributed the cellular pathophysiology of DS symptoms to the intracellular accumulation of oxidative and metabolic stressors, leading to accelerated aging [4,5,6]. Chromosome 21 encodes several genes linked to processes resulting in oxidative and metabolic stress, including mitochondrial oxidative phosphorylation (*ATP5O*, *ATP5J*, *NDUFV3*, *GABPA*) and peptide synthesis (*MRPL39*, *MRPS6*), amyloid-β generation (*APP*, *BACE*) and the repression of cellular antioxidant response pathways (*BACH1*, *RCAN1*/*DSCR1*) [7,8]. In particular, an imbalance in the activities of two genes on the duplicated chromosome 21 have been linked to decreased cell health—cystathionine-β-synthase (CBS) and copper/zinc superoxide dismutase 1 (SOD1).

It has been suggested that the triplication of the *CBS* gene locus in DS cells impairs cellular viability via the generation of excess hydrogen sulfide (H_2_S), and it has been linked to neurocognitive decline in DS mouse models [9,10,11,12]. CBS catalyzes the first step in the reverse transsulfuration pathway by converting homocysteine to cystathionine, a sulfur-containing intermediate that is subsequently cleaved by cystathionine-γ-lyase (CTH) to cysteine [10,13]. Together with 3-mercaptopyruvate sulfurtransferase (MPST), CBS and CTH are endogenous sources of H_2_S, a gaseous signaling mediator with concentration-dependent effects [10,13,14]. At lower concentrations, H_2_S is an essential signaling molecule that regulates mitochondrial energy metabolism and activates intracellular antioxidant pathways [2,15]. At higher concentrations, however, H_2_S exhibits cytotoxicity by inhibiting complex IV of the mitochondrial electron transport chain, greatly decreasing the efficiency of oxidative energy metabolism and concurrently increasing the generation of reactive oxygen species (ROS) [2,10,13,14,15,16]. The combined effects of oxidative and metabolic stress as a result of excess CBS-mediated H_2_S overproduction are thus believed to hamper viability and contribute to cellular dysfunction in DS.

In addition to CBS, the triplication of the *SOD1* locus is also associated with decreased cell health in DS. SOD1 is a major component of the cellular defense against reactive oxygen species (ROS) and catalyzes the dismutation of the superoxide radical to dioxygen and hydrogen peroxide (H_2_O_2_) [4,17]. In healthy cells, H_2_O_2_ is quickly decomposed to water and dioxygen by catalase (CAT) and peroxiredoxins. In contrast, an excess of SOD1 relative to these detoxification pathways increases the intracellular H_2_O_2_ levels in DS, which is in turn a precursor to the generation of the more damaging hydroxyl radical via the Fenton reaction [4,17,18]. Via excess H_2_O_2_ generation, SOD1-mediated oxidative stress may negatively affect DS cell health and accelerate cellular aging through cytotoxic consequences such as DNA damage and membrane lipid peroxidation [4,5,17,19,20].

In contrast to SOD1′s canonical actions with respect to superoxide radical (O_2_^●−^), SOD1 also oxidizes H_2_S to polysulfides (HS_n_^2−^), thiosulfate (S_2_O_3_^2−^) and sulfate (SO_4_^2−^) via a copper-dependent redox cycle similar to that of superoxide dismutation [21,22]. Oxidized sulfur species lack the mitochondrial toxicity of H_2_S and are also radical-scavenging antioxidants [16]. It is thus possible that SOD1 overexpression may mitigate the extent of toxic H_2_S accumulation within the DS cell and thus serve as a means of cytoprotection as well. Increased H_2_S oxidation in DS is supported by the results of prior clinical investigations by Kamoun et al., which have indicated that DS patients exhibited elevated urinary levels of the H_2_S oxidation product thiosulfate (S_2_O_3_^2−^) as well as plasma levels of sulfhemoglobin [23,24]. In addition to indicating a multisystemic program of H_2_S overproduction, these findings indicate that increased H_2_S oxidation may be an overlooked feature of DS’s molecular physiology. Further, an imbalance between H_2_S overproduction and oxidation would thus be expected to correlate negatively with cellular health in DS. 

Our group has previously reported that B lymphocytes from DS donors proliferated significantly slower than cells from apparently healthy, euploid individuals (AHIs), albeit with notable variability in the growth rates [21]. Slower B cell proliferation is consistent with the clinical presentation of lymphocytopenia observed among individuals with DS [25,26,27]. As the B cells also exhibited significantly higher levels of H_2_S and polysulfides, we suspected that the variability in DS B cell growth rates was related to phenotypic variation in H_2_S overproduction relative to AHI cells. In this report, we thus hypothesize that there exists phenotypic variation in higher basal levels of hydrogen sulfide production due to elevated CBS expression in DS B lymphocytes, leading to cytotoxic consequences. We also hypothesize that SOD1 overexpression in DS has dual consequences for B cell health: promoting cellular resilience via the removal of excess intracellular H_2_S while also contributing to an ROS burden with harmful consequences.

## 2. Methods

Cell Culture: EBV-immortalized human B lymphocytes from AHI and DS donors were obtained from the Coriell Institute for Medical Research (Camden, NJ, USA). The following naming conventions are used for these cell lines:

AHI A5522: GM25522 (F, 37 y.o.)

AHI A6363: GM16363 (M, 4 y.o.)

AHI A1954: GM01954 (F, 44 y.o.)

AHI A4569: GM14569 (M, 24 y.o.)

AHI A4592: GM14592 (M, 41 y.o.)

DS D0317: AG10317 (F, 37 y.o.)

DS D9394: AG09394 (M, 2 y.o.)

DS D7485: AG17485 (F, 44 y.o.)

DS D1921: GM01921 (M, 23 y.o.)

DS D9802: AG09802 (M, 41 y.o.)

Cells were grown in 25 mL upright flasks at 37 °C and 5% CO_2_ in 10 mL of culture media consisting of RPMI-1640 (Thermo Fisher, Waltham, MA, USA; cat. #11875093), 15% fetal bovine serum (Avantor, Radnor Township, PA, USA; cat. #97068-085) and 1% penicillin–streptomycin antibiotic cocktail. 

Measurement of Intracellular Hydrogen Sulfide and Polysulfides: For the measurement of hydrogen sulfide as previously described [21], B lymphocytes were incubated with 50 μM 7-azido-4-methylcoumarin (AzMC; λ_Ex._/λ_Em._: 360/450 nm; Cayman Chemical, Ann Arbor, MI, USA; cat. #28307) in serum- and phenol red-free culture medium for 2 h at 37 °C. To measure the intracellular polysulfides, cells were incubated with 25 μM sulfane sulfur probe 4 (SSP4; λ_Ex._/λ_Em._: 490/515 nm; Dojindo Molecular Technologies, Rockville, MD, USA; cat. #SB10) and 25 μM cetyltrimethylammonium bromide for 2 h. The cells were subsequently imaged using fluorescence microscopy under the 20× objective (Leica Microsystems DMi8; Leica Microsystems, Wetzlar, Germany). The intracellular fluorescence was quantified using ImageJ version 1.54f (National Institutes of Health, Bethesda, MD, USA).

Measurement of Intracellular Hydrogen Peroxide: To measure the intracellular hydrogen peroxide, B lymphocytes were incubated with 2.5 μM Oxivision Green (λ_Ex._/λ_Em._: 490/515 nm; AAT Bioquest, Pleasanton, CA, USA; cat. #21505) in serum- and phenol red-free culture medium for 90 min at 37 °C. After incubation, cells were centrifuged at 150 g for 6 min and pellet-reconstituted in phosphate-buffered saline. After a rest period of 30 min, the cells were visualized using fluorescence microscopy under the 20× objective. The intracellular fluorescence was quantified using ImageJ (National Institutes of Health, Bethesda, MD, USA).

Measurement of Lipid Peroxidation: B lymphocytes were incubated with 2 μM C11-BODIPY fluorophore (Invitrogen, Carlsbad, CA, USA; cat. #D3861) for 30 min at 37 °C. The cells were visualized using fluorescence microscopy under the 20× objective using the red (λ_Ex._/λ_Em._: 560/590 nm) and green (λ_Ex._/λ_Em._: 490/515 nm) fluorescence channels to measure the reduced and oxidized fluorophore signal, respectively. The intracellular fluorescence was quantified for each channel separately using ImageJ (National Institutes of Health, Bethesda, MD, USA).

Pharmacological inhibition of SOD1: Stock solutions of the SOD1 inhibitors 4,5-dichloro-2-(3-methylphenyl)-3(*2H*)-pyridazinone (LCS-1; Cayman Chemical, Ann Arbor, MI, USA; cat. #35231) and bis(choline) tetrathiomolybdate(ATN-224; Cayman Chemical, Ann Arbor, MI, USA; cat. #23533) prepared by dissolving each in dimethyl sulfoxide. Cells were treated by incubation with 15 μM LCS-1 or 10 μM ATN-224 in serum- and phenol red-free culture medium for 2 h.

Protein Immunoblotting: First, 5 × 10^6^ cells were collected and centrifuged at 2000 *g* at 4 °C for 6 min. To assess the effect of pleozyme treatment on the protein expression, B lymphocytes were administered a single dose of 4 μg/mL pleozymes or phosphate-buffered saline in culture medium and grown over a 4-day interval prior to cell collection. The supernatant was discarded, and the pellet was reconstituted in phosphate-buffered saline and centrifuged again at 2000× *g* at 4 °C for 6 min. The pellet was reconstituted in 50 μL lysis buffer, comprised of 2× RIPA buffer (Millipore Sigma, Burlington, MA, USA; cat. #20-188), 1 mM phenylmethylsulfonyl fluoride (Thermo Scientific, Waltham, MA, USA; cat. #36978), 1 mM sodium orthovanadate (Sigma-Aldrich, St. Louis, MO, USA; cat. #S6508) and 1X Halt^TM^ Protease Inhibitor Cocktail (Thermo Scientific, Waltham, MA, USA; cat. #78429). The reconstituted pellets were kept on ice and vortexed every 10 min for 30 min total, following which they were centrifuged at 16,000 rpm for 20 min at 4 °C. The supernatants were collected and measured for protein concentration using the MicroBCA^TM^ Protein Assay Kit (Thermo Scientific, Waltham, MA, USA; cat. #23235). Next, 20 µg of protein was loaded in each well of a Bolt^TM^ Bis/Tris 10% Mini Protein Gel (Thermo Scientific, Waltham, MA, USA; cat. #NW00105BOX) and separated using gel electrophoresis. The proteins were transferred onto a nitrocellulose membrane and stained for the following protein targets with primary antibodies (1:1000 dilution) for 15 h:

- CBS: rabbit anti-human CBS IgG (Abcam, Waltham, MA, USA; cat. #ab140600)
- SOD1: rabbit anti-human SOD1 IgG (Abcam, Waltham, MA, USA; cat. #ab51254)
- MPST: rabbit anti-human MPST IgG (Abcam, Waltham, MA, USA; cat. #ab154514)
- CAT: rabbit anti-human CAT IgG (Abcam, Waltham, MA, USA; cat. #ab209211)
- SQOR: anti-SQRDL antibody produced in rabbit (Sigma-Aldrich, St. Louis, MO, USA; cat. #HPA017079)

The membranes were subsequently stained with horseradish peroxidase (HRP)-conjugated goat anti-rabbit IgG (Invitrogen, Carlsbad, CA, USA; cat. #31460) for 2 h and visualized using SuperSignal^TM^ Chemiluminescence Substrate (Thermo Scientific, Waltham, MA, USA; cat. #34580). The band intensities were normalized to that of β-actin (1:10000 dilution; HRP-conjugated β-actin monoclonal antibody; Proteintech, Rosemont, IL, USA; cat. #HRP-60008). The band intensities were quantified using densitometry in ImageJ (National Institutes of Health, Bethesda, MD, USA).

Synthesis of Pleiotropic Oxidized Carbon Nanozymes (Pleozymes): Pleozymes were synthesized from the fuming nitric acid-mediated oxidation of coconut-derived, medical-grade activated charcoal as previously described [21,28,29]. In brief, activated charcoal (0.506 g) and fuming (90%) nitric acid (50 mL) were heated in a 250 mL round-bottom flask to 100 °C under reflux for 6 h. The reaction mixture was quenched by cooling over ice and purified by bath dialysis using a 1 kDa molecular weight cutoff cellulose membrane over the course of 7 days. The resulting solution of oxidized activated charcoal nanozymes (cOAC) was filtered through a 0.22 μm polyethersulfone (PES) membrane and lyophilized. Attachment of poly(ethylene glycol) (PEG) groups was achieved by a carbodiimide coupling reaction with 5 kDa methoxy-PEG-amine. The carbon core concentrations of the resulting aqueous solutions of pleozymes was determined using UV-Vis spectrophotometry (Agilent 8453 UV/Vis Spectrophotometer; Agilent Technologies, Santa Clara, CA, USA).

Measurement of Total Intracellular Oxidative Stress: Cells were incubated with 4 μg/mL pleozymes or phosphate-buffered saline in culture medium for 3 h at 37 °C and 5% CO_2_. The cells were centrifuged at 150× *g* for 6 min, supernatant discarded and pellet reconstituted in serum- and phenol red-free culture medium. Subsequently, each solution of cells was incubated with 2 μM CellROX Deep Red (Invitrogen, Carlsbad, CA, USA; cat. #C10422) for 45 min at 37 °C. The cells were visualized using fluorescence microscopy under the 20× objective using a far-red wavelength filter (λ_Ex._/λ_Em._: 630/665 nm). The intracellular fluorescence was quantified using ImageJ (National Institutes of Health, Bethesda, MD, USA).

Measurement of SOD1 Aggregation: In a procedure adapted from that of Malik et al. [30], a reaction mixture was prepared with 40 μM bovine erythrocyte SOD1 (Sigma-Aldrich, St. Louis, MO, USA; cat. #S7571), 15 μM LCS-1 and 40 μM thioflavin T (Cayman Chemical, Ann Arbor, MI, USA; cat. #32553) in phosphate-buffered saline. Then, 100 µL of the reaction mixture was loaded per well in a 96-well plate and the fluorescence readings were measured (λ_Ex._/λ_Em._: 420/480 nm) every 15 min over a 48 h interval using a BMG CLARIOstar Microplate Reader (BMG Labtech GmbH, Ortenburg, Germany) under shaking at 300 rpm and at 37 °C. 

Analysis of ATN-224 Hydrogen Sulfide Release: Solutions of ATN-224 at different concentrations and 50 μM AzMC were prepared in phosphate-buffered saline, after which 100 μL of each solution was loaded per well in a 96-well plate. The fluorescence readings (λ_Ex._/λ_Em._: 360/450 nm) were measured every 5 min over a 2 h interval using a BMG CLARIOstar Microplate Reader at 37 °C.

Statistical Analysis and Graphical Visualization: All the statistical analyses and visualizations were performed using R 4.4.1 (R Foundation for Statistical Computing, Vienna, Austria) using the tidyverse [31], rstatix [32] and ggplot2 [33] packages. The slopes of the linear regression analyses were computed using Pearson’s correlation coefficient (*r*) after checking for the normality of the residuals. Comparisons of the cohort means (AHI versus DS) were assessed for statistical significance using the *t*-test, prior to which the group mean values were tested for normality via Shapiro–Wilk tests. Comparisons of the intervention effects within cells from different individuals were assessed using the paired *t*-test if the within-individual differences in each group were considered normal, or the paired Wilcoxon signed-rank test otherwise. All the statistical analyses were tested against two-tailed null hypotheses and significance was determined when *p* < 0.05.

## 3. Results

### 3.1. H_2_S Levels in DS Cells Negatively Correlated with Growth Rate but Not Correlated with CBS Protein Levels

**Figure 1:**
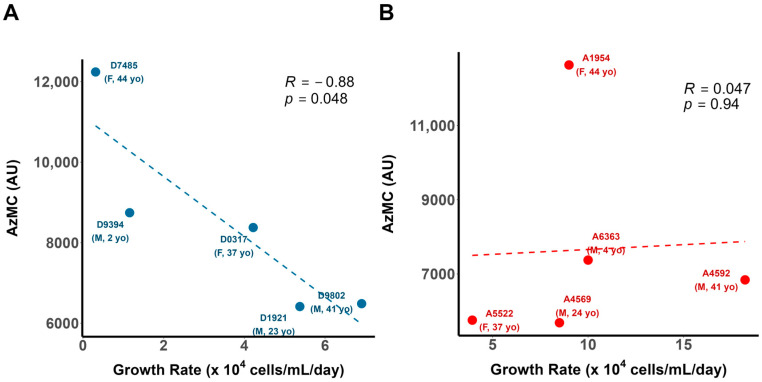
Intracellular H_2_S negatively correlated with cell growth rate in DS but not AHI B lymphocytes. Intracellular levels of hydrogen sulfide (H_2_S), measured by 7-azido-4-methylcoumarin (AzMC) fluorescence (**A**), negatively correlated with mean growth rates of DS B lymphocytes from 5 separate individuals marked as dots, measured over a 6-day interval. In contrast, (**B**) intracellular H_2_S is not significantly correlated with AHI B lymphocyte growth rates in this sample, suggesting that H_2_S levels are associated with differentially decreased cellular viability in DS. n = 5 DS and 5 AHI individuals; Pearson’s *r*.

To determine if the variability in H_2_S production was associated with decreased cellular viability in DS, we assessed the relationship between B lymphocyte growth rates and intracellular H_2_S. Using the H_2_S-selective fluorophore 7-azido-methylcoumarin (AzMC), we quantified the mean intracellular H_2_S levels for each AHI and DS cell line via fluorescence microscopy [21,34] and correlated the intracellular H_2_S values against the cell line growth rates. The intracellular H_2_S levels correlated negatively with the growth rates in DS (Figure 1A) but not the AHI B lymphocytes (Figure 1B), with considerable variability in the H_2_S levels observed within both cohorts. The significant negative association between H_2_S and the cell growth rate (r = −0.88; *p* = 0.048;) supports the existence of a maladaptive paradigm of H_2_S metabolism with consequences for lymphocyte viability in DS.

**Figure 2:**
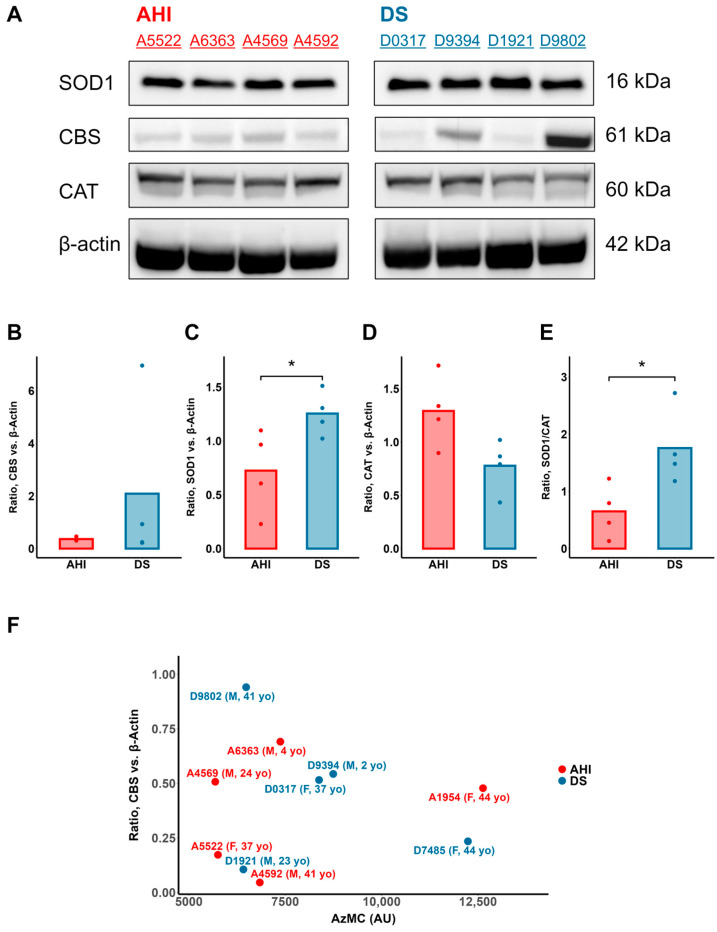
SOD1, but not CBS or catalase (CAT), consistently overexpressed in DS relative to AHI B lymphocytes. CBS levels are not correlated with intracellular H_2_S in AHI or DS B lymphocytes. (**A**) Immunoblot of relative protein levels of CBS, SOD1 and CAT in B lymphocytes from 4 AHI and 4 DS individuals, marked as dots. (**B**) CBS protein levels (normalized to β-actin) exhibited wide variability in DS B lymphocytes versus AHI, albeit not consistently overexpressed in this sample. (**C**) Normalized SOD1 protein levels significantly higher across DS B cells relative to AHI, while (**D**) protein levels of catalase trend non-significantly toward lower levels in DS. (**E**) Elevated ratio of SOD1 to CAT protein levels in DS cells, suggesting an increase in the generation of the reactive oxygen species precursor hydrogen peroxide relative to its decomposition, potentially contributing to an oxidative stress burden in DS. (**F**) CBS protein levels did not significantly correlate with intracellular levels of hydrogen sulfide (7-azido-4-methylcoumarin (AzMC) fluorescence) in B lymphocytes from AHIs (red dots) or individuals with DS (blue dots). This suggests that CBS protein levels alone may not be sufficient to explain H_2_S overproduction in DS cells. n = 4 AHI and 4 DS individuals, *t*-test; * *p* < 0.05.

We tested the hypothesis that the cytotoxic H_2_S in DS cells is due to the overexpression of CBS using protein immunoblot analyses of whole-cell lysate from each AHI and DS B cell line. The CBS expression among the DS B cell lines was higher, with two lines expressing CBS protein at levels greater than the AHI mean (DS cohort mean normalized band intensity: 2.08; AHI: 0.36). However, we did not observe significant overexpression of the protein levels in DS B cells relative to those of AHI B cells (*p* = 0.33), even though there was considerable variability in the DS CBS levels (Figure 2A,B). We found no significant correlation between CBS protein and either the intracellular H_2_S levels (Figure 2F) or cell growth rate (Figure S1). These results support the existence of phenotypic variability in intracellular H_2_S and its potential relationship with decreased cellular health in DS cells. Yet these observations also suggest that neither the cytotoxic H_2_S burden in DS cells nor decreased cell health is explained by the CBS protein levels alone. 

### 3.2. SOD1 Is an H_2_S-Oxidizing Enzyme in AHI and DS B Cells

In contrast to CBS, we observed that SOD1 protein levels were significantly elevated in DS B cells relative to their respective AHI controls (Figure 2A,C). Furthermore, the ratio of the mean SOD1 immunoblot band intensity of the DS cohort versus AHI was 1.73:1, which is close to the ratio of 1.5:1 expected because of gene triplication. We tested the hypothesis that SOD1 promotes the detoxification of excess toxic H_2_S to oxidized sulfur metabolites in DS by incubating with 15 μM of the SOD1 inhibitor 4,5-dichloro-2-m-tolylpyridazin-3(2H)-one (LCS-1) and subsequently visualized with AzMC and sulfane sulfur probe 4 (SSP4) to analyze the intracellular levels of H_2_S and polysulfides, respectively. Here, 15 µM LCS-1 was selected due to its lack of apparent cytotoxicity and 40% inhibition of bovine SOD1 superoxide dismutation in our hands and as previously reported [35]. 

The basal levels of H_2_S and polysulfides were observed to be higher in DS relative to AHI cells, albeit not significantly. Interestingly, as a function of the donor age, a trend toward decreased basal H_2_S in AHI was observed (Figure S2A), but not in DS (Figure S2B), while increased basal polysulfides in both AHI (Figure S2C) and DS (Figure S2D) were observed. 

**Figure 3:**
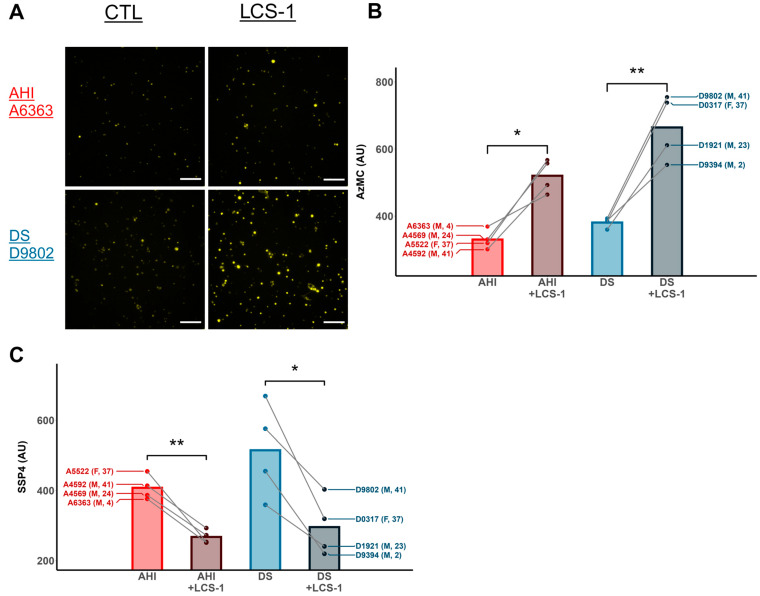
Inhibition of SOD1 with LCS-1 increased intracellular H_2_S and decreased polysulfide levels in AHI and DS B lymphocytes. (**A**) AzMC fluorescence micrographs of untreated AHI and DS B cells and cells treated with 15 μM of the SOD1 inhibitor LCS-1 for 2 h. Scale bar: 100 µm. (**B**) Incubation of AHI and DS B cells from 4 separate individuals each (represented by dots) with LCS-1 increased intracellular H_2_S levels and (**C**) decreased intracellular polysulfide levels, as measured by sulfane sulfur probe (SSP4) fluorescence relative to untreated cells, suggesting that SOD1 oxidizes H_2_S to polysulfides in this cell model. Lines connect the mean fluorescence values of untreated and LCS-1-treated cells from the same individual. n = 4 AHIs and 4 individuals with DS; paired *t*-test. * *p* < 0.05, ** *p* < 0.01.

LCS-1 increased the intracellular H_2_S levels in AHI and DS B cells relative to untreated cells (Figure 3A,B), and the magnitude of this increase was greater in the DS cohort (Figure S3). Furthermore, LCS-1 increased the intracellular H_2_S levels in a dose-dependent manner up to 15 µM in an AHI and DS B cell line (Figure S4), which may reflect a decreasing cellular capacity to oxidize H_2_S with increasing SOD1 inhibition. In contrast, LCS-1-treated AHI and DS B cells exhibit significantly reduced intracellular polysulfide levels (Figure 3C). These results suggest that SOD1 functions as an H_2_S-oxidizing enzyme in DS cells. 

**Figure 4:**
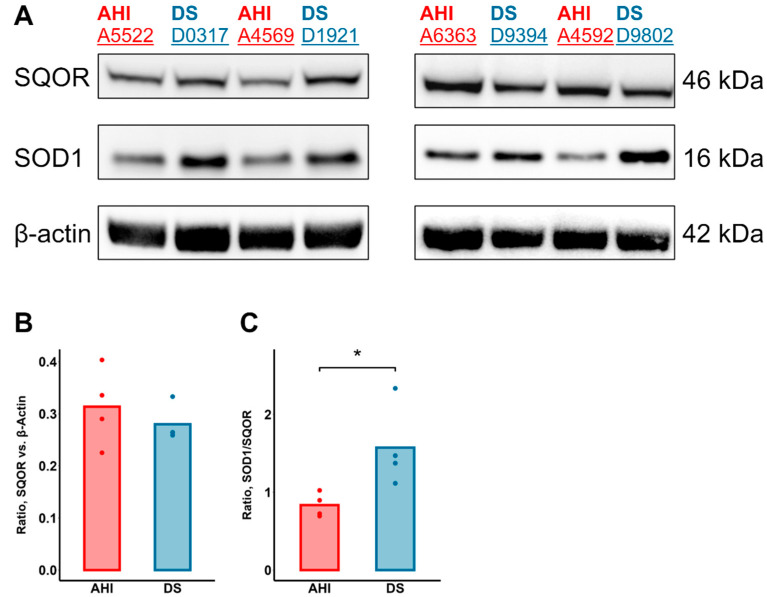
Elevated SOD1: SQOR ratio in DS B lymphocytes. (**A**) Immunoblot of relative protein levels of SQOR and SOD1 in B lymphocytes from 4 AHIs and 4 individuals with DS, marked as dots. (**B**) SQOR protein levels were not significantly different in AHI versus DS B cells, though (**C**) a higher ratio of SOD1 to SQOR protein levels was observed in the latter. This observation suggests that higher basal polysulfide levels in DS B cells versus AHI may relate more with increased SOD1 protein overexpression in comparison to the canonical (SQOR-catalyzed) H_2_S oxidation pathway. n = 4 AHIs and 4 individuals with DS, *t*-test; * *p* < 0.05.

Importantly, the levels of the prototypical H_2_S-oxidizing enzyme sulfide:quinone oxidoreductase (SQOR) were not significantly different between AHI and DS cells (Figure 4A,B). A lack of differentially upregulated SQOR expression in DS cells combined with the higher intracellular H_2_S levels observed in DS B cells after SOD1 inhibition suggests that the removal of excess intracellular H_2_S is mainly meditated by SOD1 in DS B cells. Increased non-canonical, SOD1-mediated H_2_S oxidation relative to the canonical SQOR-mediated route is further supported by the significantly higher SOD1:SQOR ratio in DS B cells (Figure 4C).

A caveat concerning the use of LCS-1 is that while its dose-dependent inhibition of SOD1 has been characterized, the precise mechanism of this action is unknown [30,36]. As LCS-1 is structurally different from copper ion chelators that decrease SOD1 activity, such as bis(choline) tetrathiomolybdate (ATN-224) [30], it is unlikely that LCS-1 functions similarly. Instead, it is possible that LCS-1 may form adducts with SOD1 that facilitate its aggregation and loss of enzymatic function. We explored the possibility of LCS-1-mediated SOD1 aggregation by monitoring changes in the fluorescence of thioflavin T—a fluorophore sensitive to β-sheet structures in protein aggregates—in the presence of bovine erythrocyte SOD1 and 15 μM LCS-1. The recurrent spikes of increased thioflavin T fluorescence intensity of SOD1 treated with LCS-1 (Figure S5) relative to untreated protein suggest the repeated formation and dissolution of SOD1 protein aggregates in the presence of LCS-1. This pattern is in contrast to the monotonic increase in thioflavin T fluorescence observed with tris(2-carboxyethyl) phosphine—a reducing denaturant that induces the irreversible aggregation of SOD1 [30]—and the fluctuations in fluorescence with LCS-1 suggest that the nature of this aggregation is more transient. Future experiments will be needed to determine the effect of LCS-1-mediated aggregation on SOD1’s superoxide dismutation and H_2_S oxidation activities. Another caveat concerning the interpretation of these results is that LCS-1 can directly oxidize H_2_S in cell-free systems [35], suggesting that our present analysis may underestimate the increase in intracellular H_2_S using this method of SOD1 inhibition. 

Given these issues with LCS-1, we tested another mode of SOD1 inhibition—ATN-224—to measure the effects of SOD1 inhibition on intracellular H_2_S levels. As ATN-224’s active tetrathiomolybdate anion is an H_2_S-releasing agent at high concentrations in aqueous solutions, we first tested whether the dose used to treat cells did not significantly increase the H_2_S levels in solution by itself and confirmed no increase in H_2_S with 10 μM ATN-224 (Figure S6). AHI and DS cells treated with 10 µM ATN-224 also exhibited increased intracellular H_2_S levels relative to untreated cells (Figure S7), consistent with our findings with LCS-1.

### 3.3. SOD1 Overexpression Contributes to an Oxidative Stress Burden in DS B Cells

**Figure 5:**
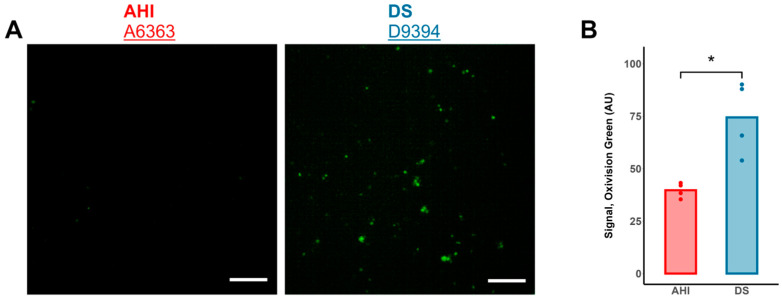
Elevated intracellular H_2_O_2_ in DS B lymphocytes. (**A**) Representative Oxivision Green micrographs of B cells from an AHI and DS individual. Scale bar: 100 µm (**B**) B lymphocytes from 4 different individuals with DS exhibited higher intracellular levels of hydrogen peroxide (H_2_O_2_), measured using the fluorophore Oxivision Green, as compared to cells from 4 different AHIs (represented by dots). n = 4 AHIs and 4 individuals with DS, *t*-test; * *p* < 0.05.

Despite its potential role in H_2_S detoxification, SOD1 overexpression in DS may have consequences for B cell health. In contrast to SOD1, protein levels of catalase were not concurrently overexpressed and trended toward lower levels across the DS cohort (Figure 2D). Therefore, there existed a significantly higher SOD1:catalase protein ratio in DS B cells relative to AHI (Figure 2E). As CAT is essential for the decomposition of excess hydrogen peroxide (H_2_O_2_) generated by SOD1 [37], an increased SOD1:catalase ratio suggests increased H_2_O_2_ generation relative to degradation in DS cells. Indeed, DS B cells exhibited significantly higher intracellular H_2_O_2_ levels relative to AHI, as measured via fluorescence microscopy with the specific fluorophore Oxivision Green (Figure 5A,B). 

**Figure 6:**
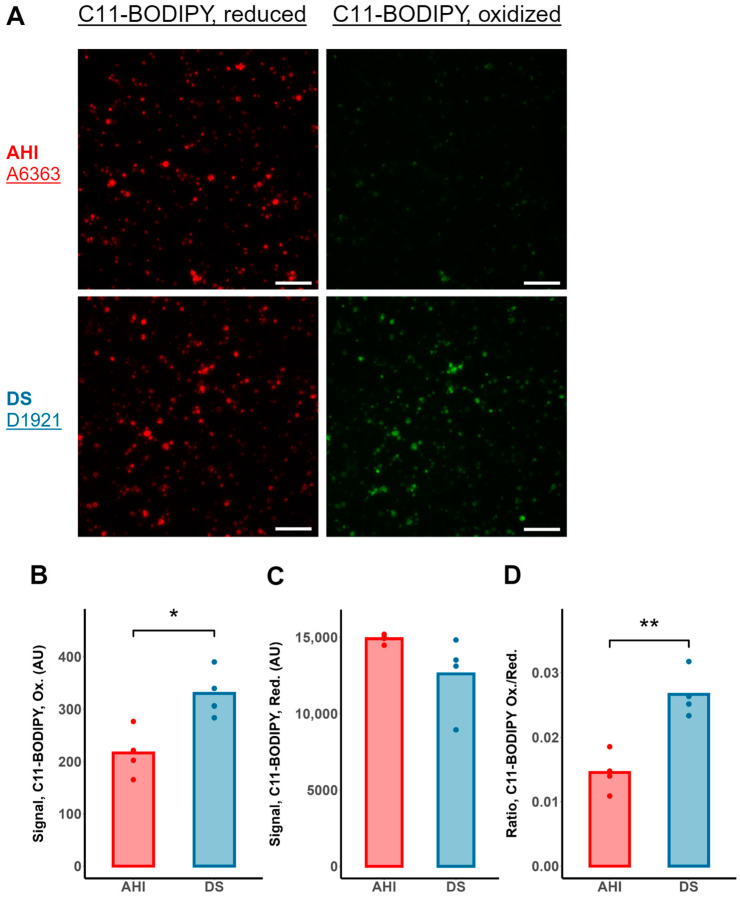
Increased lipid peroxidation in DS B lymphocytes. Quantification of lipid peroxidation in B lymphocytes from 4 different AHIs and individuals with DS (represented by dots) using C11-BODIPY, a ratiometric, lipophilic fluorophore. (**A**) Representative fluorescence micrographs of reduced (red) and oxidized (green) C11-BODIPY in B cells from an AHI and an individual with DS. Scale bar: 100 µm. (**B**) Significantly higher levels of oxidized fluorophore and (**C**) a trend toward lower fluorescence levels of reduced fluorophore, resulting in a (**D**) higher ratio of oxidized to reduced C11-BODIPY in DS B lymphocytes relative to AHI, suggesting increased levels of lipid peroxidation, a cytotoxic consequence of reactive oxygen species generation. n = 4 AHIs and 4 individuals with DS, *t*-test; * *p* < 0.05, ** *p* < 0.01.

While the toxicity of H_2_O_2_ itself is limited, it serves as the precursor to the more damaging hydroxyl radical via the Fenton reaction [18], which can subsequently attack biomolecules within the cell. A deleterious consequence of hydroxyl radical formation is the peroxidation of lipids, causing a loss of organelle and plasma membrane integrity that culminates in cell death [38]. We tested the extent of lipid peroxidation with C11-BODIPY, a fluorescence-based ratiometric probe of membrane lipid peroxidation. The fluorescence intensities of the reduced probe trended toward lower values, whereas those of the oxidized probe were significantly increased in DS B cells relative to AHI (Figure 6A–C). As such, the ratio of oxidized to reduced C11-BODIPY was also significantly elevated in the DS B cells (Figure 6D). Together, these findings suggest that SOD1 overexpression may contribute to a cytotoxic paradigm of increased oxidative stress mediated by increased generation of H_2_O_2_.

### 3.4. Ameliorative Effects of Pleozymes on Oxidative Stress Burden, MPST Levels in DS B Cells

**Figure 7:**
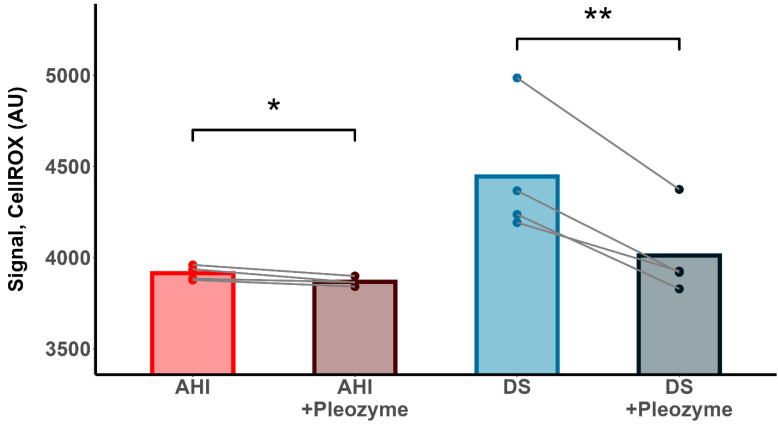
Pleozymes decreased intracellular oxidative stress in AHI and DS B lymphocytes. The total intracellular oxidative stress was measured using the fluorophore CellROX. DS B lymphocytes from 4 different individuals (represented by dots) exhibited a higher basal CellROX fluorescence versus AHI B lymphocytes, suggesting a higher accumulation of reactive oxygen species. AHI and DS B lymphocytes incubated with pleozymes for 3 h exhibited lower fluorescence levels relative to untreated cells and to a greater magnitude in DS cells, suggesting that pleozyme treatment may decrease the total burden of oxidative stress in DS cells. Lines connect the mean fluorescence values of untreated and pleozyme-treated cells from the same individual. n = 4 AHIs and 4 individuals with DS; paired *t*-test. * *p* < 0.05, ** *p* < 0.01.

The contrast between SOD1′s role as an H_2_S-oxidizing enzyme and its dysregulated generation of H_2_O_2_ suggests that its overexpression has dual consequences for the DS B cell. On the one hand, SOD1 overexpression may confer resilience against excess H_2_S; yet on the other, it may drive hydroxyl radical-mediated damage to cellular components. As such, there exists a need to balance SOD1′s potential cytoprotective effects with its consequences, which may be achieved via the application of pleiotropic oxidized carbon nanozymes, which we term “pleozymes”. In previous characterizations, we have shown that pleozymes both catalytically oxidize H_2_S and dismutate superoxide radicals, while also quenching hydroxyl radical, the latter by a presently unknown mechanism [28,39,40]. As we have previously demonstrated the utility of pleozymes in decreasing intracellular H_2_S and increasing polysulfides in DS B cells [21], we wished to investigate their ability to mitigate the total reactive oxygen species burden using CellROX Deep Red, a fluorescent indicator of hydroxyl and superoxide radicals [2]. Pleozyme treatment decreased the CellROX fluorescence intensities in AHI and DS B cells, with a greater magnitude in the latter (Figure 7). This result suggests that pleozymes can increase cellular resilience to an excess of reactive oxygen species in DS B cells, thereby limiting this toxic consequence of SOD1 overexpression.

**Figure 8:**
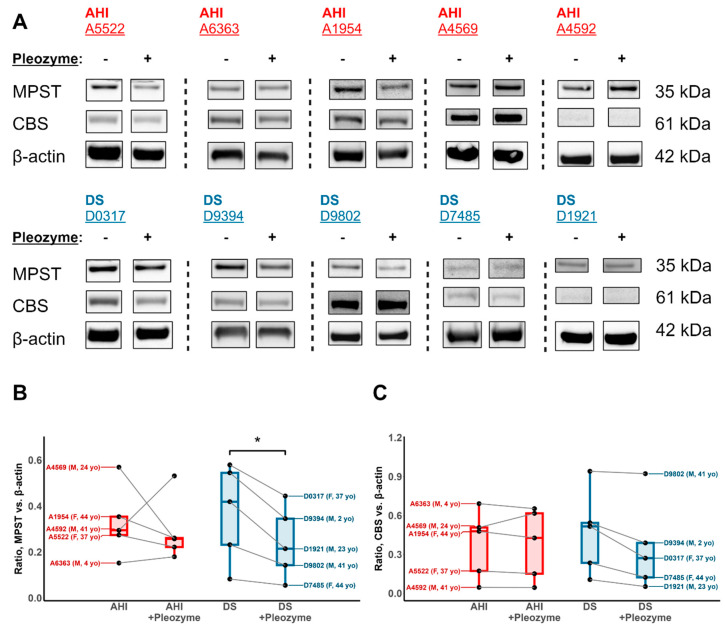
Pleozyme treatment decreases levels of MPST and CBS protein in DS B lymphocytes. (**A**) Immunoblot of MPST and CBS protein levels across B lymphocytes from 5 AHIs and 5 individuals with DS. Treatment of DS B lymphocytes with a single dose of 4 μg/mL pleozymes over a 4-day interval (**B**) significantly decreased MPST and (**C**) induced a trend toward decreased CBS protein levels, albeit not significant, relative to untreated cells. A lack of consistent trend in MPST or CBS protein levels with pleozyme treatment was observed for cells from AHIs. This observation may represent a pleozyme-mediated method of downregulating levels of H_2_S production in DS cells. Lines connect the normalized MPST immunoblot band intensities of untreated and pleozyme-treated cells from the same individual. n = 5 AHIs and 5 individuals with DS; paired *t*-test. * *p* < 0.05.

In addition to their promise in decreasing cellular oxidative stress, we posited that pleozymes may mediate other protective actions via interactions with H_2_S-generating pathways such as reverse transsulfuration and cysteine catabolism in DS cells. Whereas CBS is a major H_2_S-generating enzyme in the former pathway, MPST is recognized as a ubiquitously expressed H_2_S and polysulfide-generating enzyme in the latter [16]. To investigate the effects of pleozyme treatment on the expression of CBS and MPST, AHI and DS B cells were incubated with 4 µg/mL pleozymes for 4 days, a dose based on the previously characterized in vivo and in vitro efficacy [21,29,39,40,41,42]. Following treatment, the cell lysate was collected and analyzed via protein immunoblotting for relative target expression. The treatment significantly decreased the MPST protein levels (Figure 8A,B) and induced a trend toward decreased CBS (Figure 8C) after 4 days in DS, but not AHI B, cells. These results suggest that in addition to their beneficial H_2_S-oxidizing actions, pleozymes may downregulate key H_2_S-generating enzymes in DS B cells, a promising method of increasing cellular health that motivates future biochemical investigations.

## 4. Discussion

We show here that the phenotype of excess intracellular H_2_S exhibits considerable variability across B lymphocytes from individuals with DS and is negatively correlated with the growth rate, a parameter of cell health. These observations are consistent with the model of H_2_S-induced metabolic stress and cell growth impairment first proposed by Kamoun et al. [10,13,23,24]. However, the genomic overabundance of *CBS* on chromosome 21 was postulated as the cause of H_2_S overproduction and decreased cellular viability in DS, which we did not confirm here in B cells. Studies from other groups indicate that CBS-mediated H_2_S overproduction impairs mitochondrial bioenergetics in DS patient-derived fibroblasts and disrupts neurobehavioral function in a DS mouse model, and that these deficits were rescued after the pharmacological inhibition of CBS with aminoxyacetic acid (AOAA) or gene silencing with anti-CBS siRNA [10,11,12]. Yet we observed neither consistent CBS protein overexpression in DS cells nor a relationship between the CBS protein levels and H_2_S. The former observation indicates that *CBS* triplication does not mandate an overexpression of protein. Our observation of variability in CBS protein expression is corroborated by a recent investigation into the mRNA expression of chromosome 21 genes in individuals with DS by Donovan et al., which found variable and inconsistent overexpression of gene targets relative to euploid controls [25]. It is possible that with the analysis of a larger sample size of DS B cells, a trend toward CBS overexpression might be observed. 

Additionally, the gene location of the other major endogenous sources of H_2_S outside of chromosome 21 may not necessarily exclude them from H_2_S overproduction in DS. Notably, increased MPST-mediated H_2_S generation has been previously documented in DS fibroblasts with negative consequences for mitochondrial energy metabolism [10]. Therefore, increased MPST activity may represent another basis for H_2_S overproduction in DS cells as a consequence of increased cysteine degradation [14,16,43]. Cysteine is unique among the proteinogenic amino acids as it is also an integral component of glutathione and thioredoxin, cofactors for antioxidant enzymes that ameliorate the consequences of ROS-induced oxidative damage to DNA, proteins and membrane lipids [44]. Future experiments are needed to elucidate the relative contributions of cysteine catabolism (via MPST) and reverse transsulfuration (via CBS and CTH) to H_2_S overproduction in DS.

In contrast to CBS, SOD1 protein overexpression was observed in DS cells at a level consistent with genomic trisomy, which is consistent with prior observations by other groups of SOD1 overexpression across several cell types in human patients and DS mouse models [4,19,45]. The regularity of SOD1 overexpression across multiple cell types in DS has been interpreted as a fundamental source of oxidative stress by virtue of excess H_2_O_2_ generation in DS, but it is possible that SOD1 may serve a protective role within the disease context as well. 

While increased CBS and MPST activity has been observed to contribute to the excess H_2_S phenotype, the dynamics of H_2_S catabolism in DS are less understood. H_2_S is canonically oxidized in mitochondria to polysulfides by sulfide:quinone oxidoreductase (SQOR) and to sulfite (SO_3_^2−^) and S_2_O_3_^2−^ by persulfide dioxygenase (ETHE1) and rhodanese (TST) [27]. Considering the rapid binding kinetics of H_2_S to mitochondrial complex IV [46], it is likely that an auxiliary mechanism of H_2_S detoxification is active in DS cells in order to prevent imminent cell death [22]. Furthermore, Panagaki et al. have reported that ETHE1 and TST protein levels exist at a similar level in AHI and DS human dermal fibroblasts, potentially indicating a need for further H_2_S-oxidizing capacity in DS cells [12]. The SOD1-catalyzed oxidation of H_2_S is a compelling adjunct to the SQOR-ETHE1-TST system, considering its ubiquitous expression in the nucleus, cytoplasm and mitochondrial intermembrane space, in addition to its rapid reaction rate of sulfur oxidation [22,35,47]. SOD1 protein overexpression in DS may thus limit H_2_S overproduction through the rapid generation of oxidized sulfur species, and conversely, the loss of SOD1 activity would thus be expected to increase the difference in intracellular H_2_S levels between DS and AHI cells, as observed in this report. Our finding of decreased intracellular polysulfides upon pharmacological inhibition of SOD1 in DS and AHI cells further supports this conclusion, although it must be noted that SOD1 can oxidize H_2_S to sulfate as well [22]. As our method for polysulfide detection is not sensitive to other oxidized sulfur species such as thiosulfate, sulfite and sulfate, it is possible that the results of this report underestimate the true extent of SOD1-mediated H_2_S oxidation. A key limitation of the methods of pharmacological SOD1 inhibition used in this report is the possibility of off-target effects not due to decreased SOD1 activity [48]. Future work using specific genetic tools to selectively ablate SOD1 activity would thus be important to confirm the results presented here.

Despite the apparently protective role of SOD1 as an H_2_S-oxidizing enzyme, our observation of significantly higher intracellular H_2_O_2_ levels indicates that SOD1 overexpression may also increase the ROS burden in DS cells. SOD1-mediated H_2_O_2_ generation is enabled by increased mitochondrial generation of superoxide anion in DS as a result of inefficient oxidative phosphorylation and dysregulated mitochondrial biogenesis, themselves consequences of trisomy 21 [49,50]. Ironically, the “detoxification” of the superoxide radical to H_2_O_2_ generates the precursor to the more damaging hydroxyl radical via the Fenton reaction, for which there does not exist an enzymatic mechanism of elimination [18,38]. The peroxidation of membrane lipids is a particularly harmful manifestation of hydroxyl radical toxicity and is implicated in the premature aging of neurons in DS animal models [4,20,51]. Our observation of increased lipid peroxidation is thus consistent with prior characterizations in other DS models and may indicate that SOD1 overexpression may directly contribute to an oxidative stress burden in DS.

**Figure 9:**
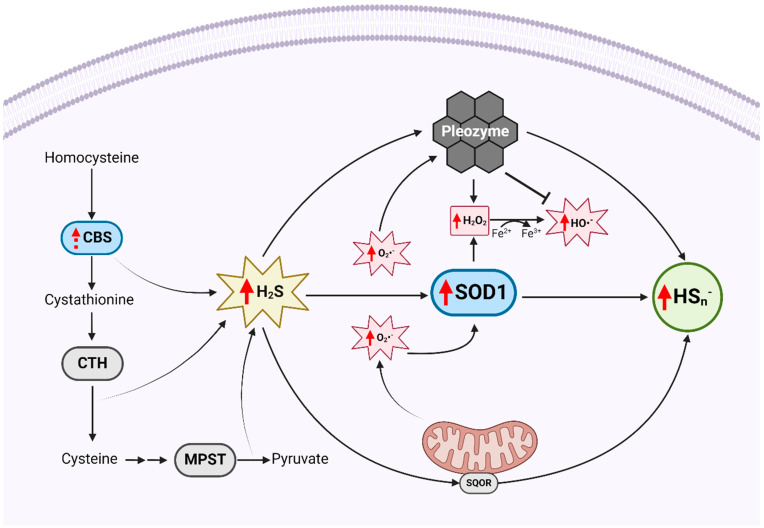
Schematic of protective and deleterious activities of SOD1 on hydrogen sulfide (H_2_S) and reactive oxygen species (ROS) metabolism in Down syndrome (DS). Red vertical arrow indicates that a species or protein is elevated in DS relative to AHI B lymphocytes, dashed arrow indicates a trend toward elevation. Blue shading indicates proteins encoded on chromosome 21. Excess H_2_S is produced in DS B lymphocytes by the combined activities of cystathionine-β-synthase (CBS), cystathionine-γ-lyase (CTH) and 3-mercaptopyruvate sulfurtransferase (MPST). While H_2_S is canonically oxidized to polysulfides (HS_n_^−^) through mitochondrial sulfide:quinone oxidoreductase (SQOR), it is also oxidized by superoxide dismutase 1 (SOD1). SOD1 overexpression in DS may therefore confer protection to DS cells against H_2_S, but at the cost of increasing the intracellular ROS burden through the dismutation of excess superoxide anion (O_2_*^−^) to hydrogen peroxide (H_2_O_2_), a precursor to the harmful hydroxyl radical (HO*^−^). By oxidizing H_2_S and quenching ROS, pleozymes may augment the cytoprotective role of SOD1 while mitigating the oxidative consequences of its overexpression in DS B lymphocytes. Created in BioRender. Mouli, K. (2024) BioRender.com/r68r888 (accessed on 10 January 2024).

Our results therefore support the existence of an intertwined relationship between dysregulated sulfide and reactive oxygen species metabolism in DS, with negative consequences for cell health and proliferation (Figure 9). Intriguingly, we observed that the intracellular polysulfide levels in AHI and DS B lymphocytes increased as a function of the donor age, a trend also reported by Ikeda et al. in plasma samples from apparently healthy humans of a comparable age range (22–43 years) [52]. We also observed a trend toward decreased intracellular H_2_S in AHIs with age, which, together with the increased polysulfide levels, may suggest that H_2_S directly reacts with the higher levels of ROS that occur with aging. Increased ROS generation leading to oxidative hepatic injury and depletion of reduced glutathione stores as a function of age is a noteworthy phenomenon in DS mouse models [53]. Therefore, it is possible that increased oxidation of H_2_S to polysulfides is a cytoprotective mechanism that counters aging-exacerbated oxidative stress via polysulfide-mediated ROS scavenging and regeneration of reduced and persulfide glutathione [54].

In light of the deleterious, multisystemic oxidative stress burden in DS, several trials of chronic antioxidant therapies targeted at ameliorating oxidative stress have been previously initiated, including those of vitamin E [55,56], vitamin A [56,57], vitamin C [7,56], selenium, zinc, folinic acid [57] and α-lipoic acid [7]. However, trials of antioxidant therapies failed to exhibit substantial improvements to symptoms or quality of life [7]. Additionally, none of the aforementioned trials measured H_2_S oxidation as an endpoint for improving the parameters of cell health.

Here, we introduce a new strategy for pleiotropic H_2_S oxidation and ROS dismutation using pleozymes, thereby harnessing SOD1’s cytoprotective benefits while mitigating its potentially toxic consequences for DS cells. Pleozymes have a broad redox potential within the range of several biologically relevant substrates [21,28,39]. In particular, pleozymes can dismutate superoxide and quench hydroxyl radicals [28,40,58], oxidize H_2_S [21] and facilitate electron transfer reactions between NADH and oxidized cytochrome c, thereby enhancing cellular energy metabolism [21,29,39]. We posited that these pleiotropic actions of pleozymes could increase resilience to oxidative stress in DS cells by mitigating a major consequence of SOD1 overexpression. Our observations support a role for pleozymes as mediators of cellular health that have the potential to bridge the protective and deleterious effects of SOD1 overexpression in DS, one that merits further investigations in cell and animal models of the disease.

## Data Availability

The original contributions presented in this study are included in the article/supplementary material. Further inquiries can be directed to the corresponding authors.

## Supplementary Materials

The following supporting information can be downloaded at: https://www.mdpi.com/article/10.3390/antiox13111361/s1, Figure S1: CBS protein levels not significantly correlated with DS or AHI lymphocyte growth rates. CBS protein levels, measured by normalized immunoblot band intensities are not correlated with B lymphocyte growth rates across (A) 5 DS and (B) 5 AHI individuals. Dots represent mean value pairs from each individual. n = 5 DS and 5 AHI individuals; Pearson’s *r*. Figure S2: Influence of age. Intracellular H_2_S and age negatively correlated in AHI, lack of correlation in DS B lymphocytes, with trend toward increased polysulfides as a function of age in AHI and DS. Intracellular H_2_S, measured using 7-azido-4-methylcoumarin (AzMC) fluorescence intensity (A) negatively correlated with donor age in B lymphocytes from apparently healthy individuals (AHI), while (B) no significant association between H_2_S and age was observed in cells from Down syndrome (DS) individuals. Trend toward increased intracellular polysulfide levels as measured by sulfane sulfur probe 4 (SSP4) fluorescence in (C) AHI and (D) DS cells. n = 4 DS and 4 AHI individuals; Pearson’s *r*. Figure S3: Increase in intracellular H_2_S following SOD1 inhibition is greater in DS B cells versus AHI. DS cells incubated with the SOD1 inhibitor LCS-1 for 2 h exhibited higher intracellular hydrogen sulfide levels as a cohort relative to LCS-1-treated AHI cells. This suggests that the increase in intracellular hydrogen sulfide levels upon SOD1 inhibition is greater in DS versus AHI cells. n = 4 AHI and 4 DS individuals; *t*-test. * *p* < 0.05. Figure S4: LCS-1 and AzMC standard curve. B lymphocytes from an AHI and DS individual were incubated with different concentrations of LCS-1 for 2 h, after which intracellular hydrogen sulfide levels were measured using 7-azido-4-methylcoumarin (AzMC) fluorescence. Cells exhibit a dose-dependent increase in AzMC fluorescence, with a higher peak AzMC fluorescence occurring at a higher LCS-1 concentration in DS cells (15 μM) versus AHI (10 μM). This is consistent with the observation of higher SOD1 protein levels in DS cells versus AHI. Figure S5: LCS-1 may induce transient aggregation of SOD1. Change in fluorescence of the protein aggregation fluorophore thioflavin T in the presence of 40 μM SOD1 only, with 15 μM LCS-1 or with 50 mM of the reducing denaturant tris(2-carboxyethyl) phosphine (TCEP) over a 48-h interval. A monotonic increase in thioflavin T fluorescence with TCEP treatment indicates the formation of SOD1 aggregates over time. The prevalence of spiked increases in thioflavin T fluorescence with LCS-1 treatment may represent temporary aggregations of SOD1 and their subsequent dissolution. n = 4 technical replicates/group; mean +/− standard deviation (shaded region). Figure S6: ATN-224 does not significantly increase H_2_S levels in solution at a dose range of 5-10 µM. Changes in the fluorescence of 7-azido-4-methylcoumarin (AzMC) in solutions of 5, 10 and 50 μM bis(choline) tetrathiomolybdate (ATN-224) in phosphate-buffered saline (PBS). While 50 μM ATN-224 notably increased AzMC fluorescence over a 2-h interval, no similar increase was observed for 5 and 10 μM ATN-224 relative to PBS only. This suggests that concentrations of ATN-224 relevant to cell treatment may not affect measurements of intracellular hydrogen sulfide via AzMC fluorescence. n = 4 technical replicates/group; mean +/− standard deviation (shaded region). Figure S7: Inhibition of SOD1 with ATN-224 increases intracellular H _2_S in AHI and DS B lymphocytes. B lymphocytes from an AHI and a DS individual incubated with 10 μM of the copper chelator bis(choline) tetrathiomolybdate (ATN-224) exhibit increased intracellular hydrogen sulfide as reflected in increased 7-azido-4-methylcoumarin fluorescence. Scale bar: 100 µm.

## References

[1] Antonarakis S.E., Skotko B.G., Rafii M.S., Strydom A., Pape S.E., Bianchi D.W., Sherman S.L., Reeves R.H. (2020). Down syndrome. Nat. Rev. Dis. Primers.

[2] Celeghini E.C.C., Alves M.B.R., de Arruda R.P., de Rezende G.M., Florez-Rodriguez S.A., de Sá Filho M.F. (2021). Efficiency of CellROX deep red^®^ and CellROX orange^®^ fluorescent probes in identifying reactive oxygen species in sperm samples from high and low fertility bulls. Anim. Biotechnol..

[3] Letourneau A., Antonarakis S.E., Dierssen M., De La Torre R. (2012). Chapter 2—Genomic determinants in the phenotypic variability of Down syndrome. Progress in Brain Research.

[4] Perluigi M., Butterfield D.A. (2012). Oxidative Stress and Down Syndrome: A Route toward Alzheimer-Like Dementia. Curr. Gerontol. Geriatr. Res..

[5] Peng L., Baradar A.A., Aguado J., Wolvetang E. (2023). Cellular senescence and premature aging in Down Syndrome. Mech. Ageing Dev..

[6] Gensous N., Bacalini M.G., Franceschi C., Garagnani P. (2020). Down syndrome, accelerated aging and immunosenescence. Semin. Immunopathol..

[7] Lott I.T. (2012). Antioxidants in Down syndrome. Biochim. Biophys. Acta (BBA)-Mol. Basis Dis..

[8] Roizen N.J., Patterson D. (2003). Down’s syndrome. Lancet.

[9] Marechal D., Brault V., Leon A., Martin D., Lopes Pereira P., Loaëc N., Birling M.C., Friocourt G., Blondel M., Herault Y. (2019). Cbs overdosage is necessary and sufficient to induce cognitive phenotypes in mouse models of Down syndrome and interacts genetically with Dyrk1a. Hum. Mol. Genet..

[10] Panagaki T., Randi E.B., Szabo C. (2020). Role of 3-Mercaptopyruvate Sulfurtransferase in the Regulation of Proliferation and Cellular Bioenergetics in Human Down Syndrome Fibroblasts. Biomolecules.

[11] Panagaki T., Lozano-Montes L., Janickova L., Zuhra K., Szabo M.P., Majtan T., Rainer G., Maréchal D., Herault Y., Szabo C. (2022). Overproduction of hydrogen sulfide, generated by cystathionine β-synthase, disrupts brain wave patterns and contributes to neurobehavioral dysfunction in a rat model of down syndrome. Redox Biol..

[12] Panagaki T., Randi E.B., Augsburger F., Szabo C. (2019). Overproduction of H_2_S, generated by CBS, inhibits mitochondrial Complex IV and suppresses oxidative phosphorylation in Down syndrome. Proc. Natl. Acad. Sci. USA.

[13] Petrosino M., Zuhra K., Kopec J., Hutchin A., Szabo C., Majtan T. (2022). H_2_S biogenesis by cystathionine beta-synthase: Mechanism of inhibition by aminooxyacetic acid and unexpected role of serine. Cell. Mol. Life Sci..

[14] Pedre B., Talwar D., Barayeu U., Schilling D., Luzarowski M., Sokolowski M., Glatt S., Dick T.P. (2023). 3-Mercaptopyruvate sulfur transferase is a protein persulfidase. Nat. Chem. Biol..

[15] Kimura H., Shibuya N., Kimura Y. (2012). Hydrogen sulfide is a signaling molecule and a cytoprotectant. Antioxid. Redox Signal..

[16] Noguchi N., Saito Y., Niki E. (2023). Actions of Thiols, Persulfides, and Polysulfides as Free Radical Scavenging Antioxidants. Antioxid. Redox Signal..

[17] Eleutherio E.C.A., Silva Magalhães R.S., de Araújo Brasil A., Monteiro Neto J.R., de Holanda Paranhos L. (2021). SOD1, more than just an antioxidant. Arch. Biochem. Biophys..

[18] Lenzen S., Lushchak V.I., Scholz F. (2022). The pro-radical hydrogen peroxide as a stable hydroxyl radical distributor: Lessons from pancreatic beta cells. Arch. Toxicol..

[19] Gulesserian T., Seidl R., Hardmeier R., Cairns N., Lubec G. (2001). Superoxide Dismutase SOD1, Encoded on Chromosome 21, but Not SOD2 is Overexpressed in Brains of Patients with down Syndrome. J. Investig. Med..

[20] Ishihara K., Amano K., Takaki E., Ebrahim A.S., Shimohata A., Shibazaki N., Inoue I., Takaki M., Ueda Y., Sago H. (2009). Increased lipid peroxidation in Down’s syndrome mouse models. J. Neurochem..

[21] Derry P.J., Liopo A.V., Mouli K., McHugh E.A., Vo A.T.T., McKelvey A., Suva L.J., Wu G., Gao Y., Olson K.R. (2024). Oxidation of Hydrogen Sulfide to Polysulfide and Thiosulfate by a Carbon Nanozyme: Therapeutic Implications with an Emphasis on Down Syndrome. Adv. Mater..

[22] Switzer C.H., Kasamatsu S., Ihara H., Eaton P. (2023). SOD1 is an essential H_2_S detoxifying enzyme. Proc. Natl. Acad. Sci. USA.

[23] Belardinelli M.C., Chabli A., Chadefaux-Vekemans B., Kamoun P. (2001). Urinary sulfur compounds in Down syndrome. Clin. Chem..

[24] Kamoun P., Belardinelli M.C., Chabli A., Lallouchi K., Chadefaux-Vekemans B. (2003). Endogenous hydrogen sulfide overproduction in Down syndrome. Am. J. Med. Genet. A.

[25] Donovan M.G., Rachubinski A.L., Smith K.P., Araya P., Waugh K.A., Enriquez-Estrada B., Britton E.C., Lyford H.R., Granrath R.E., Schade K.A. (2024). Multimodal analysis of dysregulated heme metabolism, hypoxic signaling, and stress erythropoiesis in Down syndrome. Cell Rep..

[26] Donovan M.G., Eduthan N.P., Smith K.P., Britton E.C., Lyford H.R., Araya P., Granrath R.E., Waugh K.A., Enriquez Estrada B., Rachubinski A.L. (2024). Variegated overexpression of chromosome 21 genes reveals molecular and immune subtypes of Down syndrome. Nat. Commun..

[27] Landry A.P., Ballou D.P., Banerjee R. (2021). Hydrogen Sulfide Oxidation by Sulfide Quinone Oxidoreductase. Chembiochem.

[28] McHugh E.A., Liopo A.V., Mendoza K., Robertson C.S., Wu G., Wang Z., Chen W., Beckham J.L., Derry P.J., Kent T.A. (2024). Oxidized Activated Charcoal Nanozymes: Synthesis, and Optimization for In Vitro and In Vivo Bioactivity for Traumatic Brain Injury. Adv. Mater..

[29] Mouli K., Liopo A.V., McHugh E.A., Underwood E., Zhao J., Dash P.K., Vo A.T.T., Malojirao V., Hegde M., Tour J.M. (2024). Oxidized Carbon Nanoparticles Enhance Cellular Energetics With Application to Injured Brain. Adv. Healthc. Mater..

[30] Malik R., Corrales C., Linsenmeier M., Alalami H., Sepanj N., Bitan G. (2020). Examination of SOD1 aggregation modulators and their effect on SOD1 enzymatic activity as a proxy for potential toxicity. FASEB J..

[31] Wickham H., Averick M., Bryan J., Chang W., McGowan L.D.a., François R., Grolemund G., Hayes A., Henry L., Hester J. (2019). Welcome to the Tidyverse. J. Open Source Softw..

[32] Kassambara A. (2023). Rstatix: Pipe-Friendly Framework for Basic Statistical Tests.

[33] Wickham H. (2016). Ggplot2: Elegant Graphics for Data Analysis.

[34] Takano Y., Shimamoto K., Hanaoka K. (2016). Chemical tools for the study of hydrogen sulfide (H_2_S) and sulfane sulfur and their applications to biological studies. J. Clin. Biochem. Nutr..

[35] Olson K.R., Takata T., Clear K.J., Gao Y., Ma Z., Pfaff E., Mouli K., Kent T.A., Jones P., Fukuto J. (2024). The SOD1 Inhibitor, LCS-1, Oxidizes H_2_S to Reactive Sulfur Species, Directly and Indirectly, through Conversion of SOD1 to an Oxidase. Antioxidants.

[36] Somwar R., Erdjument-Bromage H., Larsson E., Shum D., Lockwood W.W., Yang G., Sander C., Ouerfelli O., Tempst P.J., Djaballah H. (2011). Superoxide dismutase 1 (SOD1) is a target for a small molecule identified in a screen for inhibitors of the growth of lung adenocarcinoma cell lines. Proc. Natl. Acad. Sci. USA.

[37] Muchová J., Žitňanová I., Ďuračková Z. (2014). Oxidative stress and Down syndrome. Do antioxidants play a role in therapy?. Physiol. Res..

[38] Ayala A., Muñoz M.F., Argüelles S. (2014). Lipid peroxidation: Production, metabolism, and signaling mechanisms of malondialdehyde and 4-hydroxy-2-nonenal. Oxid. Med. Cell. Longev..

[39] Derry P.J., Nilewski L.G., Sikkema W.K.A., Mendoza K., Jalilov A., Berka V., McHugh E.A., Tsai A.L., Tour J.M., Kent T.A. (2019). Catalytic oxidation and reduction reactions of hydrophilic carbon clusters with NADH and cytochrome C: Features of an electron transport nanozyme. Nanoscale.

[40] Samuel E.L., Marcano D.C., Berka V., Bitner B.R., Wu G., Potter A., Fabian R.H., Pautler R.G., Kent T.A., Tsai A.L. (2015). Highly efficient conversion of superoxide to oxygen using hydrophilic carbon clusters. Proc. Natl. Acad. Sci. USA.

[41] Bitner B.R., Marcano D.C., Berlin J.M., Fabian R.H., Cherian L., Culver J.C., Dickinson M.E., Robertson C.S., Pautler R.G., Kent T.A. (2012). Antioxidant carbon particles improve cerebrovascular dysfunction following traumatic brain injury. ACS Nano.

[42] Fabian R.H., Derry P.J., Rea H.C., Dalmeida W.V., Nilewski L.G., Sikkema W.K.A., Mandava P., Tsai A.L., Mendoza K., Berka V. (2018). Efficacy of Novel Carbon Nanoparticle Antioxidant Therapy in a Severe Model of Reversible Middle Cerebral Artery Stroke in Acutely Hyperglycemic Rats. Front. Neurol..

[43] Shibuya N., Tanaka M., Yoshida M., Ogasawara Y., Togawa T., Ishii K., Kimura H. (2009). 3-Mercaptopyruvate sulfurtransferase produces hydrogen sulfide and bound sulfane sulfur in the brain. Antioxid. Redox Signal..

[44] Lu S.C. (2013). Glutathione synthesis. Biochim. Biophys. Acta.

[45] Cowley P.M., Nair D.R., DeRuisseau L.R., Keslacy S., Atalay M., DeRuisseau K.C. (2017). Oxidant production and SOD1 protein expression in single skeletal myofibers from Down syndrome mice. Redox Biol..

[46] Petersen L.C. (1977). The effect of inhibitors on the oxygen kinetics of cytochrome c oxidase. Biochim. Biophys. Acta.

[47] Olson K.R., Gao Y., Arif F., Arora K., Patel S., DeLeon E.R., Sutton T.R., Feelisch M., Cortese-Krott M.M., Straub K.D. (2018). Metabolism of hydrogen sulfide (H_2_S) and Production of Reactive Sulfur Species (RSS) by superoxide dismutase. Redox Biol..

[48] Steverding D., Barcelos Y. (2020). Cytotoxic Activity of LCS-1 is not Only due to Inhibition of SOD1. Drug Res..

[49] Capone G., Kim P., Jovanovich S., Payne L., Freund L., Welch K., Miller E., Trush M. (2002). Evidence for increased mitochondrial superoxide production in Down syndrome. Life Sci..

[50] Tan K.L., Lee H.C., Cheah P.S., Ling K.H. (2023). Mitochondrial Dysfunction in Down Syndrome: From Pathology to Therapy. Neuroscience.

[51] Prutton K.M., Marentette J.O., Leifheit B.A., Esquer H., LaBarbera D.V., Anderson C.C., Maclean K.N., Roede J.R. (2022). Oxidative stress as a candidate mechanism for accelerated neuroectodermal differentiation due to trisomy 21. Free Radic. Biol. Med..

[52] Ikeda M., Ishima Y., Chuang V.T.G., Sakai M., Osafune H., Ando H., Shimizu T., Okuhira K., Watanabe H., Maruyama T. (2019). Distribution of Polysulfide in Human Biological Fluids and Their Association with Amylase and Sperm Activities. Molecules.

[53] Giallongo S., Ferrigno J., Caltabiano R., Broggi G., Alanazi A.M., Distefano A., Tropea E., Tramutola A., Perluigi M., Volti G.L. (2024). Aging exacerbates oxidative stress and liver fibrosis in an animal model of Down Syndrome. Aging.

[54] Lindahl S., Xian M. (2023). Recent development of polysulfides: Chemistry and biological applications. Curr. Opin. Chem. Biol..

[55] Petersen R.C., Thomas R.G., Grundman M., Bennett D., Doody R., Ferris S., Galasko D., Jin S., Kaye J., Levey A. (2005). Vitamin E and donepezil for the treatment of mild cognitive impairment. N. Engl. J. Med..

[56] Morris M.C., Evans D.A., Bienias J.L., Tangney C.C., Bennett D.A., Aggarwal N., Wilson R.S., Scherr P.A. (2002). Dietary intake of antioxidant nutrients and the risk of incident Alzheimer disease in a biracial community study. JAMA.

[57] Ellis J.M., Tan H.K., Gilbert R.E., Muller D.P., Henley W., Moy R., Pumphrey R., Ani C., Davies S., Edwards V. (2008). Supplementation with antioxidants and folinic acid for children with Down’s syndrome: Randomised controlled trial. BMJ.

[58] Jalilov A.S., Zhang C., Samuel E.L.G., Sikkema W.K.A., Wu G., Berka V., Kent T.A., Tsai A.-L., Tour J.M. (2016). Mechanistic Study of the Conversion of Superoxide to Oxygen and Hydrogen Peroxide in Carbon Nanoparticles. ACS Appl. Mater. Interfaces.

