# Supplementary material for: SOD1 Is an Integral Yet Insufficient Oxidizer of Hydrogen Sulfide in Trisomy 21 B Lymphocytes and Can Be Augmented by a Pleiotropic Carbon Nanozyme
Source: Antioxidants (Basel). 2024 Nov 7;13(11):1361. doi: 10.3390/antiox13111361 (PMC11591310; doi:10.3390/antiox13111361)
Supplement: Supplementary file 1 [file antioxidants-13-01361-s001.zip › antioxidants-3277451-supplementary.pdf]

A

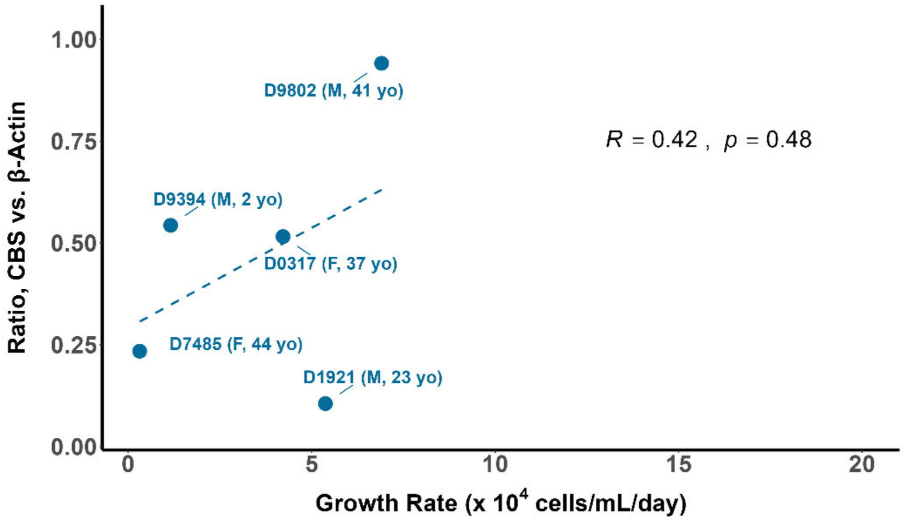

B

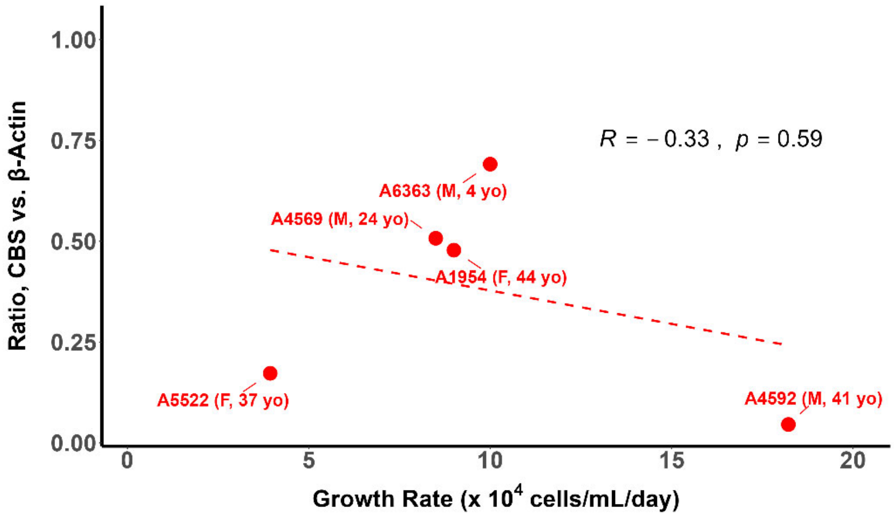

Figure S1. CBS protein levels not significantly correlated with DS or AHI lymphocyte growth rates. CBS protein levels, measured by normalized immunoblot band intensities are not correlated with B lymphocyte growth rates across (A) 5 DS and (B) 5 AHI individuals. Dots represent mean value pairs from each individual. n = 5 DS and 5 AHI individuals; Pearson's *r*.

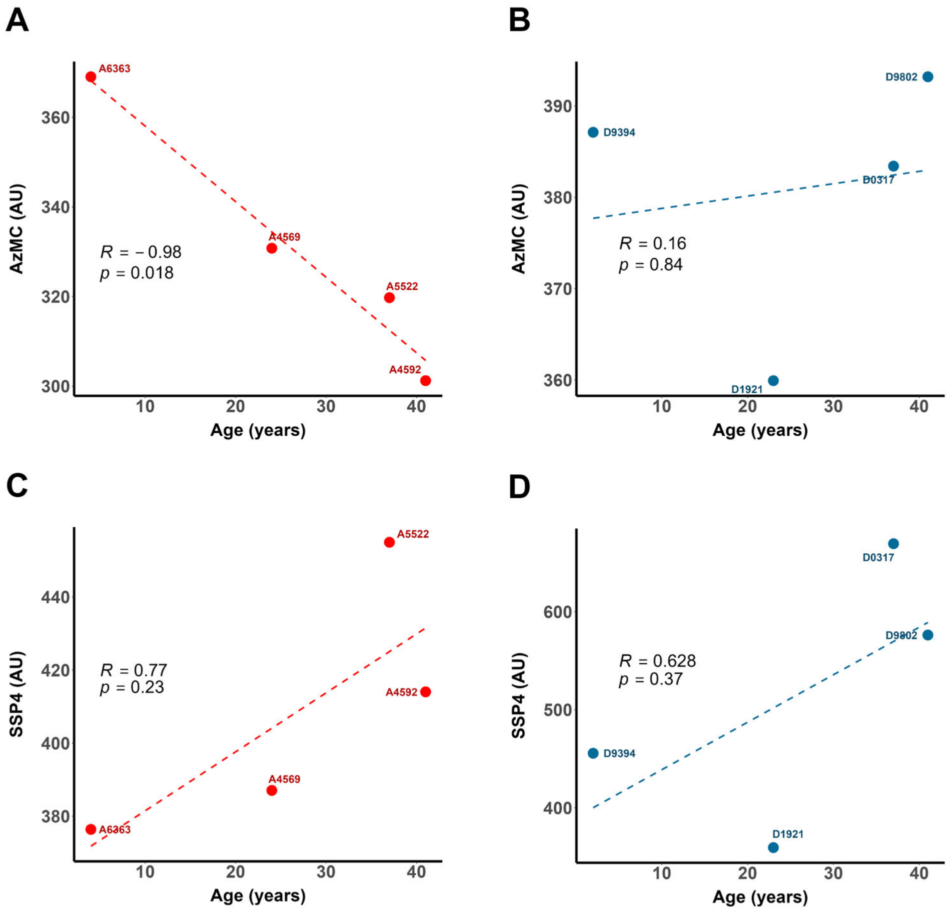

Figure S2. Influence of age. Intracellular H_2S and age negatively correlated in AHI, lack of correlation in DS B lymphocytes, with trend toward increased polysulfides as a function of age in AHI and DS. Intracellular H_2S, measured using 7-azido-4-methylcoumarin (AzMC) fluorescence intensity (A) negatively correlated with donor age in B lymphocytes from apparently healthy individuals (AHI), while (B) no significant association between H_2S and age was observed in cells from Down syndrome (DS) individuals. Trend toward increased intracellular polysulfide levels as measured by sulfane sulfur probe 4 (SSP4) fluorescence in (C) AHI and (D) DS cells. n = 4 DS and 4 AHI individuals; Pearson's *r*.

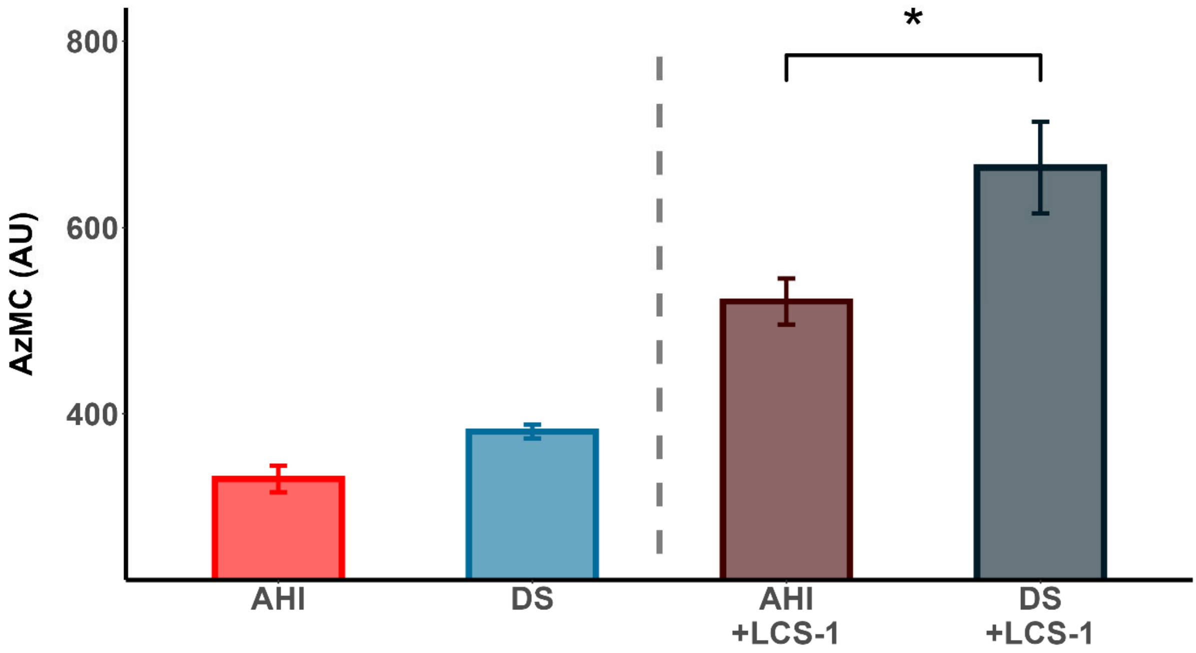

Figure S3. Increase in intracellular H_2S following SOD1 inhibition is greater in DS B cells versus AHI. DS cells incubated with the SOD1 inhibitor LCS-1 for 2 hours exhibited higher intracellular hydrogen sulfide levels as a cohort relative to LCS-1-treated AHI cells. This suggests that the increase in intracellular hydrogen sulfide levels upon SOD1 inhibition is greater in DS versus AHI cells. n = 4 AHI and 4 DS individuals; t test. * $p < 0.05$.

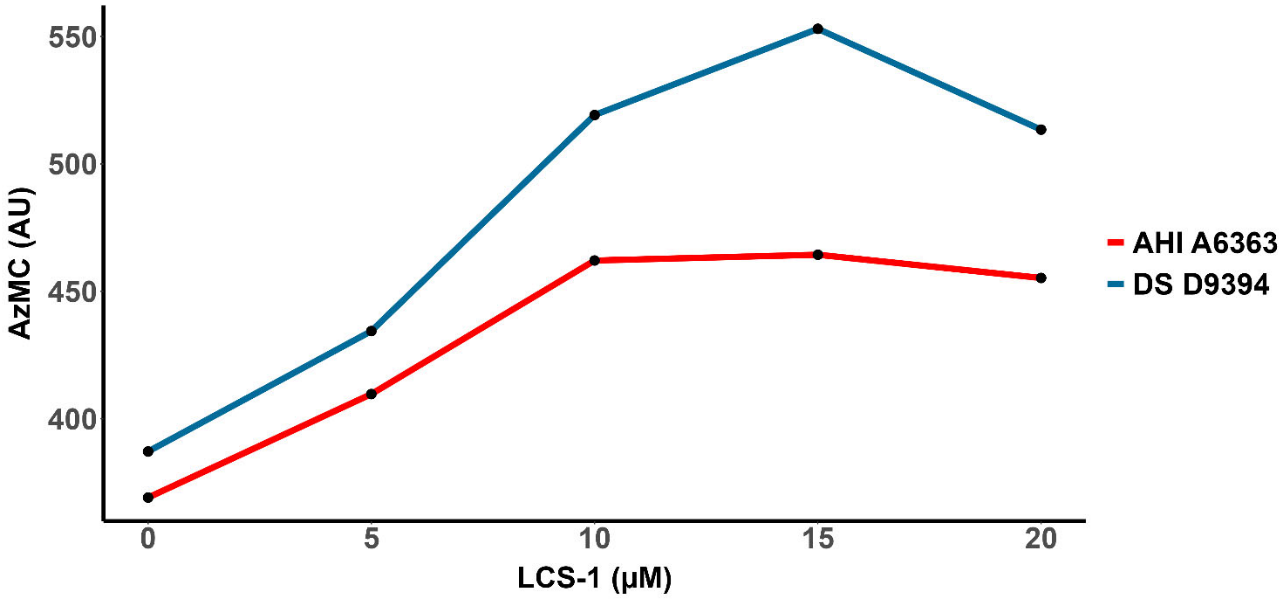

Figure S4. LCS-1 and AzMC standard curve. B lymphocytes from an AHI and DS individual were incubated with different concentrations of LCS-1 for 2 hours, after which intracellular hydrogen sulfide levels were measured using 7-azido-4-methylcoumarin (AzMC) fluorescence. Cells exhibit a dose-dependent increase in AzMC fluorescence, with a higher peak AzMC fluorescence occurring at a higher LCS-1 concentration in DS cells (15 μM) versus AHI (10 μM). This is consistent with the observation of higher SOD1 protein levels in DS cells versus AHI.

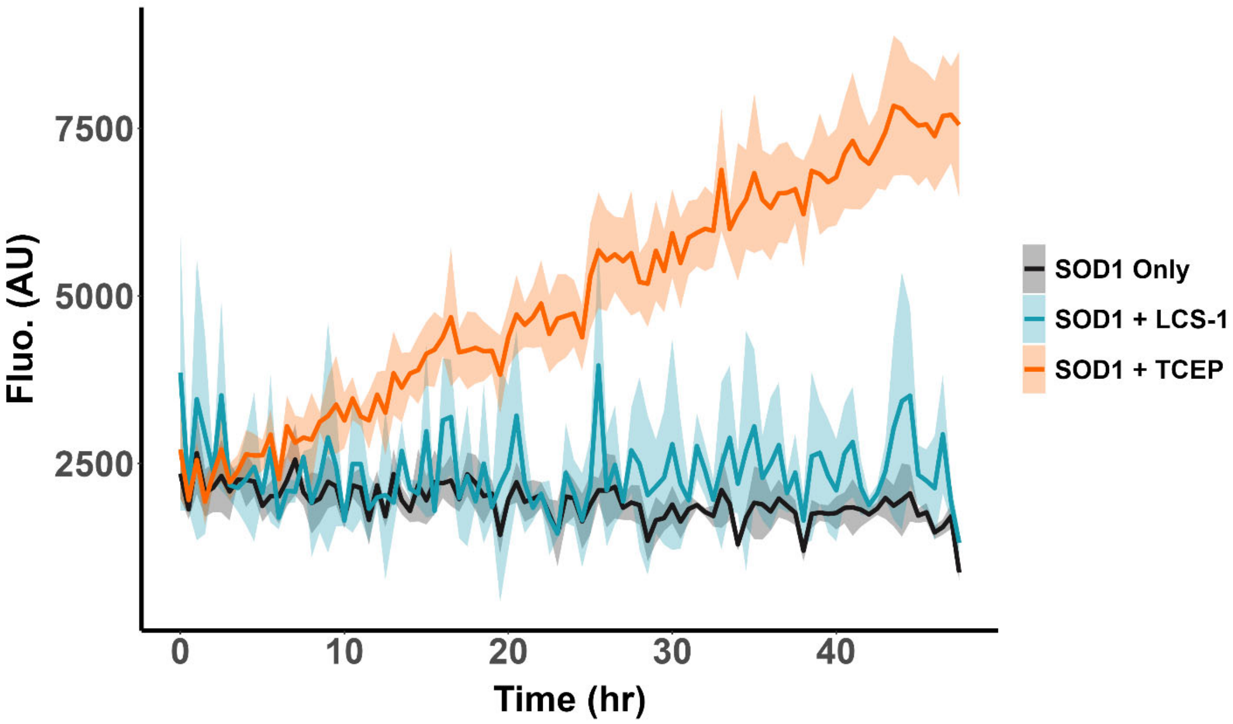

Figure S5. LCS-1 may induce transient aggregation of SOD1. Change in fluorescence of the protein aggregation fluorophore thioflavin T in the presence of 40 μM SOD1 only, with 15 μM LCS-1 or with 50 mM of the reducing denaturant tris(2-carboxyethyl) phosphine (TCEP) over a 48-hour interval. A monotonic increase in thioflavin T fluorescence with TCEP treatment indicates the formation of SOD1 aggregates over time. The prevalence of spiked increases in thioflavin T fluorescence with LCS-1 treatment may represent temporary aggregations of SOD1 and their subsequent dissolution. n = 4 technical replicates/group; mean +/- standard deviation (shaded region).

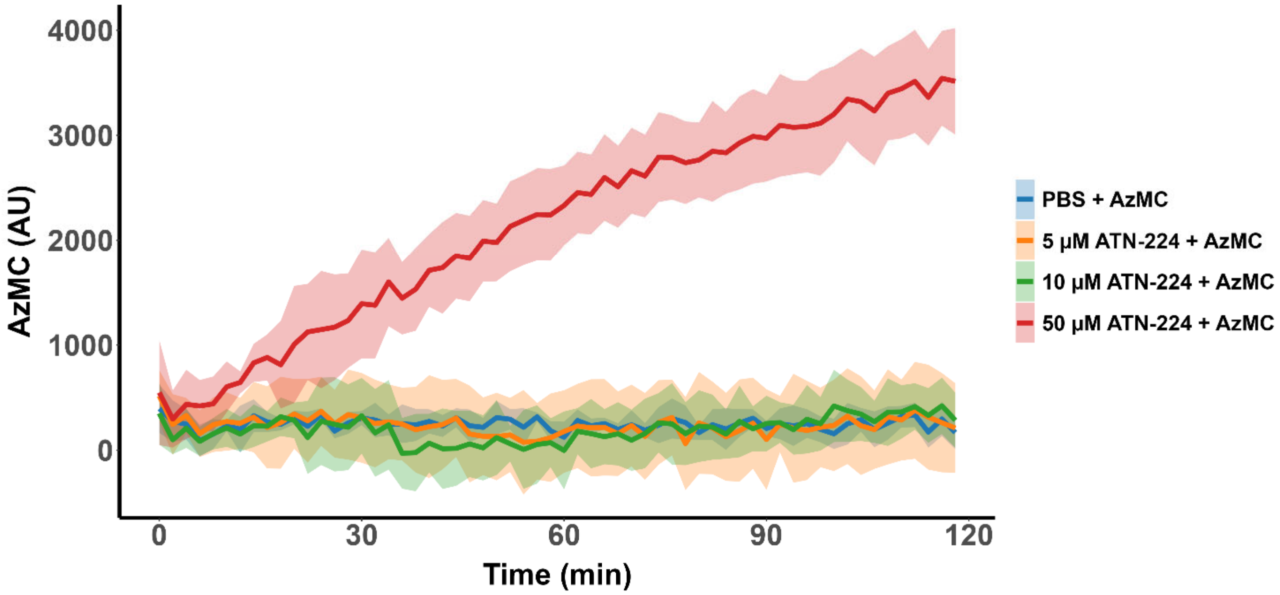

Figure S6. ATN-224 does not significantly increase H_2S levels in solution at a dose range of 5-10 μM. Changes in the fluorescence of 7-azido-4-methylcoumarin (AzMC) in solutions of 5, 10 and 50 μM bis(choline) tetrathiomolybdate (ATN-224) in phosphate-buffered saline (PBS). While 50 μM ATN-224 notably increased AzMC fluorescence over a 2-hour interval, no similar increase was observed for 5 and 10 μM ATN-224 relative to PBS only. This suggests that concentrations of ATN-224 relevant to cell treatment may not affect measurements of intracellular hydrogen sulfide via AzMC fluorescence. n = 4 technical replicates/group; mean +/- standard deviation (shaded region).

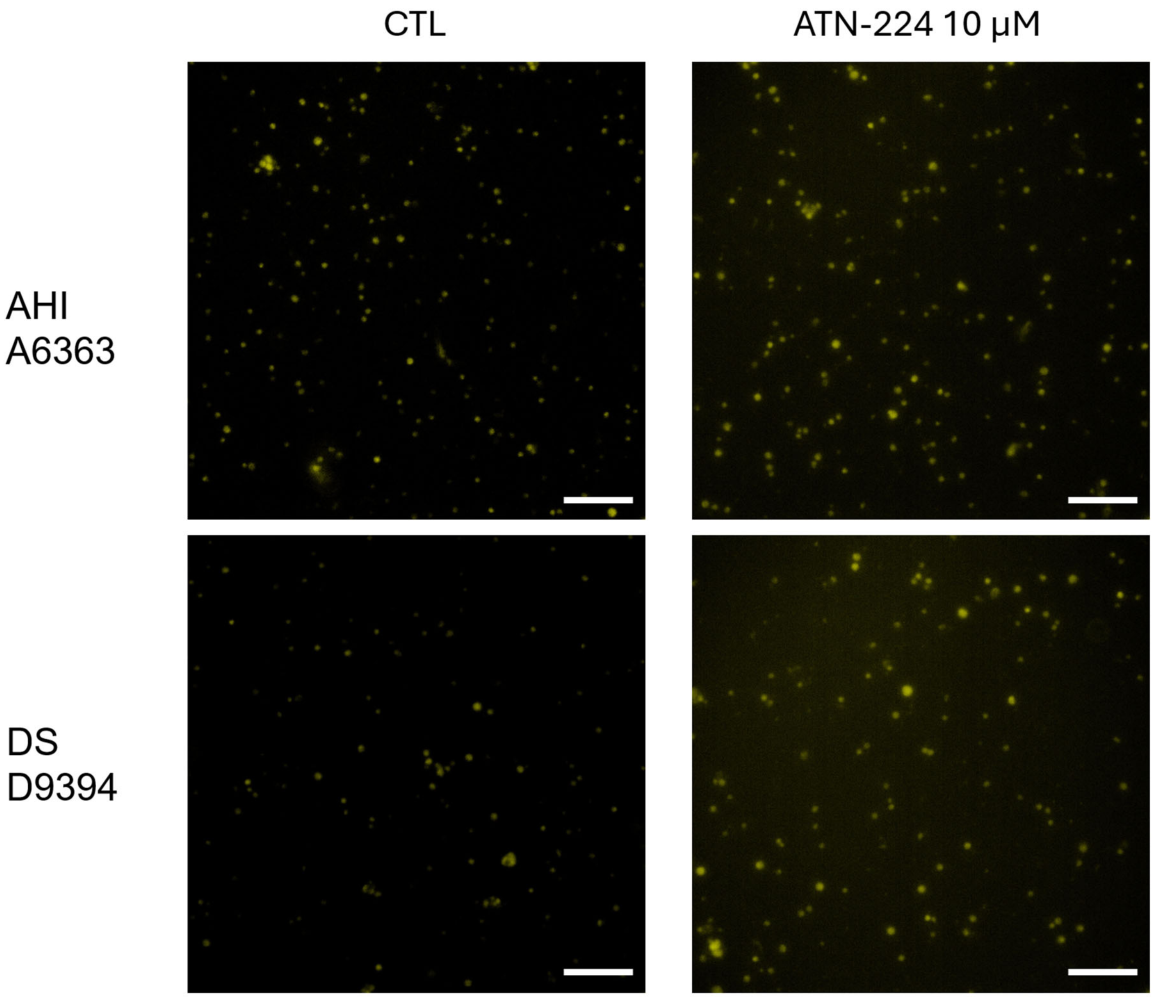

Figure S7. Inhibition of SOD1 with ATN-224 increases intracellular H_2S in AHI and DS B lymphocytes. B lymphocytes from an AHI and a DS individual incubated with 10 μM of the copper chelator bis(choline) tetrathiomolybdate (ATN-224) exhibit increased intracellular hydrogen sulfide as reflected in increased 7-azido-4-methylcoumarin fluorescence. Scale bar: 100 μm.
